# A Gaussia luciferase reporter assay for the evaluation of coronavirus Nsp5/3CLpro activity

**DOI:** 10.1038/s41598-024-71305-6

**Published:** 2024-09-05

**Authors:** Asimenia Vlachou, Rayhane Nchioua, Kerstin Regensburger, Frank Kirchhoff, Dorota Kmiec

**Affiliations:** Institute of Molecular Virology, Ulm University Medical Center, 89081 Ulm, Germany

**Keywords:** SARS-CoV-2, Coronaviruses, Gaussia reporter assay, Nsp5, 3CLpro, Protease inhibitor, SARS-CoV-2, Drug screening, Biological techniques

## Abstract

Human coronaviruses (hCoVs) infect millions of people every year. Among these, MERS, SARS-CoV-1, and SARS-CoV-2 caused significant morbidity and mortality and their emergence highlights the risk of possible future coronavirus outbreaks. Therefore, broadly-active anti-coronavirus drugs are needed. Pharmacological inhibition of the hCoV protease Nsp5 (3CLpro) is clinically beneficial as shown by the wide and effective use of Paxlovid (nirmatrelvir, ritonavir). However, further treatment options are required due to the risk of drug resistance. To facilitate the assessment of coronavirus protease function and its pharmacological inhibition, we developed an assay allowing rapid and reliable quantification of Nsp5 activity under biosafety level 1 conditions. It is based on an ACE2-Gal4 transcription factor fusion protein separated by a Nsp5 recognition site. Cleavage by Nsp5 releases the Gal4 transcription factor, which then induces the expression of Gaussia luciferase. Our assay is compatible with Nsp5 proteases from all hCoVs and allows simultaneous measurement of inhibitory and cytotoxic effects of the tested compounds. Proof-of-concept measurements confirmed that nirmatrelvir, GC376 and lopinavir inhibit SARS-CoV-2 Nsp5 function. Furthermore, the assay accurately predicted the impact of Nsp5 mutations on catalytic activity and inhibitor sensitivity. Overall, the reporter assay is suitable for evaluating viral protease activity.

## Introduction

Since 2019, the coronavirus disease 2019 (COVID-19) pandemic claimed the lives of more than seven million people and affected the health and livelihoods of almost everyone^1^. Prior to the emergence of its causative agent, severe acute respiratory syndrome coronavirus 2 (SARS-CoV-2), four other human coronaviruses (hCoVs), often referred to as common cold coronaviruses (ccCoVs; NL63, 229E, OC43 and HKU1) were endemic, and two others—severe acute respiratory syndrome (SARS-CoV-1) and Middle East Respiratory Syndrome (MERS-CoV) – caused limited epidemic outbreaks of severe respiratory diseases^2^. ccCoV infections generally do not require medical intervention in healthy individuals, but can lead to hospitalization and death in children, elderly and immunocompromised patients^3^. In comparison, SARS-CoV-1, SARS-CoV-2 and MERS-CoV infections are more severe with average case fatality rates of approximately 10%, 1% and 40%, respectively^4–6^. The repeated emergence of hCoVs in the recent decades and the speed at which SARS-CoV-2 spread globally highlight the risk of possible future outbreaks of novel zoonotic coronaviruses. Altogether, there is a need to develop effective and broadly active antiviral compounds against the present and future strains of hCoVs.

All coronaviruses have large (25–32 kb) RNA genomes, two thirds of which is taken by the open reading frame (ORF) *1ab*. The product, polyprotein pp1ab, is cleaved into 16 non-structural proteins (Nsp) by 2 viral proteases, the papain-like protease Nsp3 and the main protease Nsp5^3^. Both of these enzymes are functionally conserved among coronaviruses and are essential for their replication. Thus, they represent useful targets of antiviral therapies. Nsp3 cleaves pp1ab at 3 sites leading to Nsps 1–3, while the main protease Nsp5 cleaves at 11 sites, generating the remaining Nsps 4–16^3^. The Nsp5, commonly referred to as 3-chymotrypsin-like protease (3CLpro) catalytically cleaves a peptide bond after a glutamine residue.

Protease inhibitors that block pp1ab processing are highly efficient at limiting CoV replication, as they target an early and essential step in virus replication. Paxlovid, a combination of the two viral protease inhibitors nirmatrelvir and ritonavir, is one of the few approved and widely used treatments for COVID-19 and has been reported to show ~ 88% efficacy in preventing hospitalization or death^7^. Alarmingly, recent studies identified several mutations in the SARS-CoV-2 main protease that give rise to nirmatrelvir resistance, highlighting the need for additional anti-coronavirus compounds^8–15^. Other protease inhibitors have been considered but are not approved for COVID-19 treatment. For example, GC376 is used in veterinary medicine to treat fatal feline coronavirus infection. Lopinavir blocks protease activity and is used in anti-retroviral therapy of HIV-infected patients^16–18^ but has shown little to no benefit in the treatment of COVID-19^19^. Lufotrelvir is the phosphate prodrug of the protease inhibitor PF-00835231^19^. Lufotrelvir was investigated in pre-clinical and clinical trials against COVID-19 but was less successful than nirmatrelvir due to its low oral bioavailability and fast systemic clearance^20,21^. Additional protease inhibitors such as simotrelvir, bofutrelvir and 11d, have shown promising efficacy and tolerability in vitro and in vivo^22–25^. While still not widely available, U.S. Food and Drug Administration (FDA) has granted the Fast Track designation for an investigational COVID-19 oral antiviral ensitrelvir following its approval in Japan and Singapore^26^. Since experiments with infectious SARS-CoV-2, SARS-CoV-1, and MERS require biosafety level 3 facilities, virus-free drug screening platforms are strongly desired to foster drug discovery. Since 2020, several SARS-CoV-2 protease reporter assays have been developed^27–30^. These methods mostly rely on the quantification of the fluorescence signal produced upon Nsp5-mediated cleavage of Flip-GFP inducing its conformational change from a non-fluorescent to a fluorescent state. However, this approach requires optimization to ensure specificity and does not allow to easily distinguish between protease-specific and cytotoxicity-related decreases in cell fluorescence. GFP is also pH-sensitive and prone to photobleaching, which might introduce biases and reduce reproducibility during high-throughput drug screening. To overcome these issues, we designed a Gaussia luciferase-based Nsp5 reporter system that produces a robust signal within as little as 24 h and is compatible with the affordable and effective MTT (3-(4,5-dimethylthiazol-2-yl)-2,5-diphenyltetrazolium bromide) cell viability assay. Reporters based on secreted Gaussia luciferase offer non-invasive sampling, high stability, and compatibility with virus inactivation procedures. Thus, they represent useful tools for studying coronavirus protease activity.

## Results

### Generation of a Gaussia luciferase-based coronavirus Nsp5 activity reporter

**Fig. 1:**
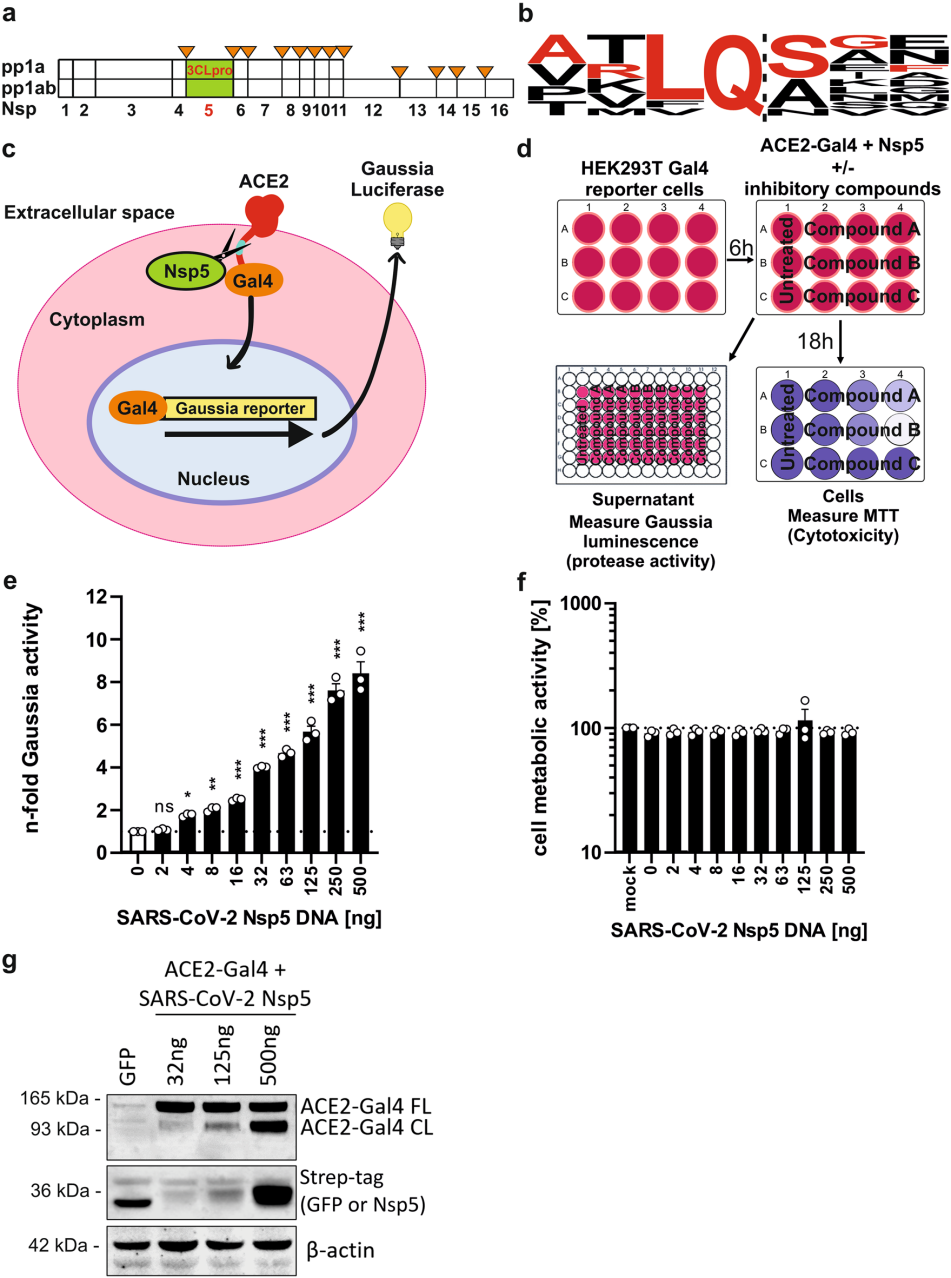
Design and performance of the Nsp5 reporter assay. (**a**) Location of Nsp5 cleavage sites in coronavirus polyprotein 1ab. (**b**) Sequence logo of the Nsp5 recognition site based on SARS-CoV-2 1ab polyprotein. The sequence of representative cleavage site selected for testing is indicated in red. (**c**) Principle of the reporter assay. Human ACE2 fused to transcription factor Gal4 is expressed in HEK293T cells containing a stable Gaussia luciferase reporter downstream of 5 × Gal4 binding sites. The fusion protein contains Nsp5 recognition site in the flexible glycine linker region. Upon cleavage, Gal4 fused to amino acids 364–550 of mouse NF-kB translocates into the nucleus where it activates the transcription of Gaussia luciferase, which is subsequently secreted into cell supernatant. (**d**) Experimental outline of the Nsp5 reporter assay. HEK293T cells containing the stable Gaussia luciferase reporter downstream of the 5 × Gal4 binding sites are co-transfected with construct encoding ACE2-Gal4 fusion protein, and varying amounts of Strep-tagged Nsp5 or GFP (negative control). At 18-20 h post transfection supernatants are harvested for luminescence measurement of Gaussia luciferase activity (relative light units per second; rlu/s) using coelenterazine substrate, while the cells are subjected to 2,5-diphenyl-2H-tetrazolium bromide (MTT) assay measuring cell metabolic activity. (**e**) Normalized n-fold increase in Gaussia luciferase activity over background (GFP + reporter only) by transfected SARS-CoV-2 Nsp5, and (**f**) corresponding MTT assay results measured at 18 h post transfection. (**g**) Western blot of transfected cell lysates showing dose-dependent ACE2-Gal4 reporter cleavage (*FL* full length, *CL* cleaved protein detected by ACE2 staining). Lane 1 contained GFP only control. Mean of 3 independent experiments + SD. *P < 0.05; **P < 0.01; ***P < 0.001, ns, P > 0.05. Unpaired Student’s *t*-test.

Nsp5 is crucial for processing of Nsps 5–16, making it an essential component of the SARS-CoV-2 replication machinery (Fig. 1a). It cleaves after a glutamine residue (Q)^3^, which is highly conserved and found at all SARS-CoV-2 Nsp5-16 borders (Fig. 1b). To exploit the specificity of Nsp5 in designing a novel coronavirus reporter assay, we generated a plasmid encoding a fusion protein of human angiotensin-converting-enzyme 2 (ACE2), and the transcription factor galactose utilization 4 (Gal4). ACE2 was selected due to its role as the major entry receptor of three human coronaviruses (SARS-CoV-1, SARS-CoV-2 and NL63). The two components were joined by a flexible G/S-linker containing the protease recognition sequence ARLQSGF. This motif resembles the naturally found cleavage site between SARS-CoV-2 Nsp4 and Nsp5 (AVLQ|SGF) but contains a single V/R substitution reported to enhance processing^27^. In the presence of active Nsp5, this fusion protein undergoes cleavage and Gal4, which contains a nuclear import signal, translocates to the nucleus, where it activates the transcription of an integrated luciferase reporter (Fig. 1c). Gaussia luciferase is released into the supernatant, where it can be detected in a non-invasive manner following the addition of its substrate coelenterazine. This method produces a stable luminescence signal, which allows to measure changes in protease activity without cell lysis. It is also compatible with MTT assay measuring cell metabolic activity, which is commonly used to evaluate cytotoxicity (Fig. 1d).

At 18 h post-transfection, the assay showed a dose-dependent increase in Gaussia luciferase activity with no signs of protease-induced cytotoxicity (Fig. 1e,f). The reporter detected as little as 4 ng of the transfected Nsp5 expression construct, indicating that it is a robust quantitative method of evaluating coronavirus protease activity. The results were highly reproducible and the highest Nsp5 gene dose (500 ng) resulted in an ~ eightfold increase in Gaussia activity over background at 18 h post-transfection (Fig. S1A). With longer incubation times the background signal increased gradually, leading to a lower signal-to-noise ratio at later time points (Fig. S1B). Therefore, 18-24 h was selected as a standard for further experiments. Dose-dependent cleavage of the ACE2-Gal4 reporter by Nsp5 was also observed as two distinct protein forms (full-length: FL and cleaved: CL) in Western blot analysis of the transfected cell lysates, although this method was less sensitive than the luciferase reporter assay and required longer incubation (Fig. 1g). Overall, the developed Nsp5 reporter was found to be an easy and rapid method of quantifying SARS-CoV-2 main protease activity.

### Comparison of the activity of Nsp5 proteins from all seven hCoVs

**Fig. 2:**
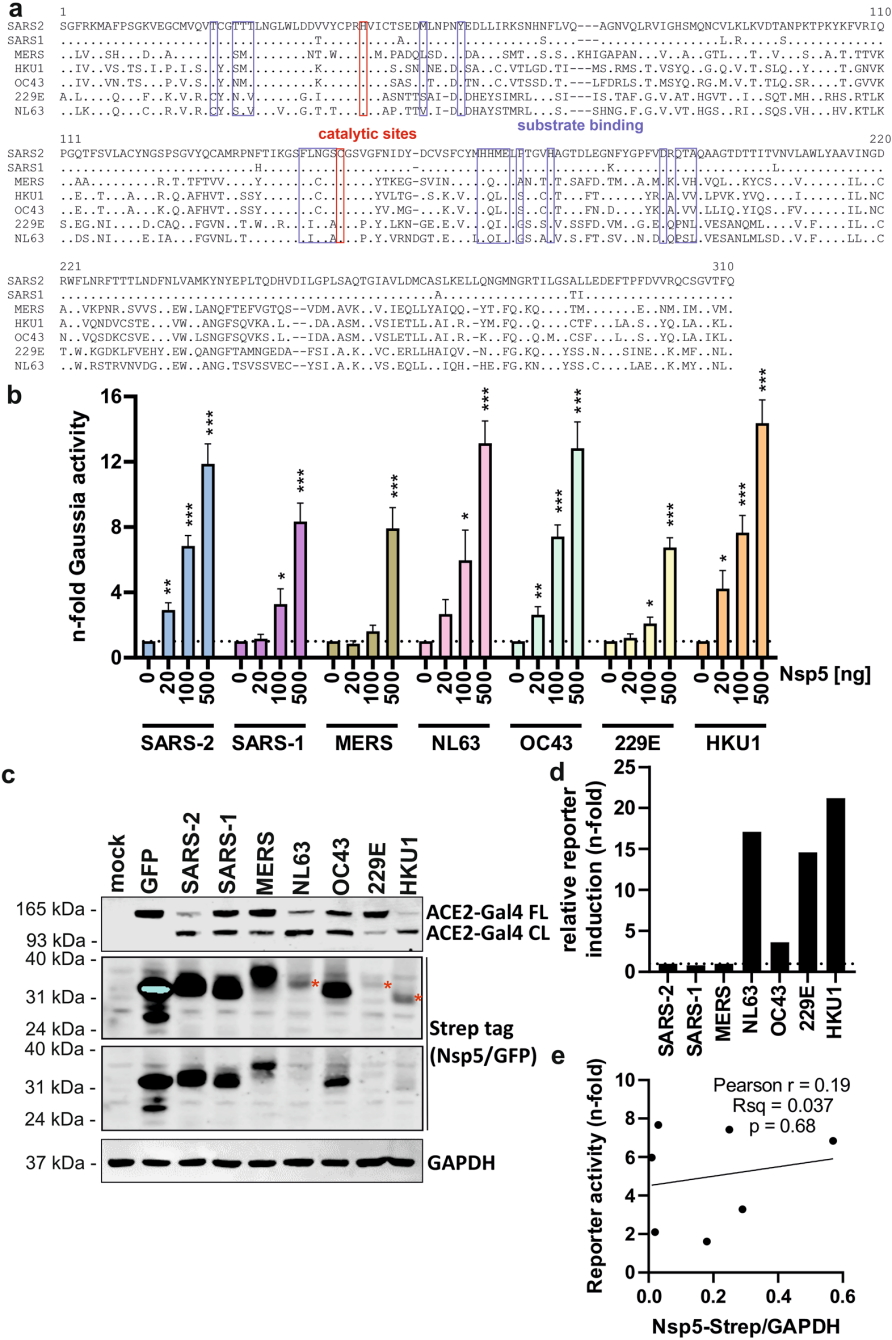
Activation of the reporter assay by hCoV Nsp5. (**a**) Amino acid sequence alignment of hCoV Nsp5 proteins. Catalytic residues are highlighted in red and substrate binding sites are indicated by blue brackets. Domain mapping was based on similarity to SARS-CoV-2 sequence. (**b**) Dose-dependent activation of the reporter assay by overexpressed hCoV Nsp5 proteins shown as n-fold enhancement over negative control (ACE2-Gal4 + GFP). Mean of 4 independent experiments + SD. *P < 0.05; **P < 0.01; ***P < 0.001, unpaired Student’s *t*-test. (**c**) Western blot of cellular lysates following co-expression of the ACE2-Gal4 reporter and indicated Strep-tagged hCoV Nsp5 proteins or GFP. GAPDH served as protein loading control. Red stars indicate the relative gel position of low expressed proteins. (**d**) Levels of Gaussia luciferase activity normalized to protein expression levels measured by a Western blot. (**e**) Lack of significant correlation between reporter activity measured in (**b**) at the 100 ng dose and Nsp5 protein expression levels observed in western blot (**c**).

The Nsp5 proteins encoded by the seven different hCoVs have conserved function and all contain H41 and C145 catalytic residues. However, these homologs share less than 50% amino acid sequence identity, with multiple changes within the reported substrate binding site^31^ (Fig. 2a). Therefore, we investigated if the developed Nsp5 reporter could detect the activity of proteases from diverse hCoVs. To address this, we synthesized expression constructs containing codon-optimized Nsp5 sequences from the seven hCoV species and co-expressed each of them with the ACE2-Gal4 reporter. Nsp5 overexpression had no effect on cell viability (Fig. S2). The expression of Nsp5 from all hCoVs led to significant and dose-dependent increases in reporter activity, with NL63 (alpha-coronavirus), OC43 and HKU1 (beta-coronaviruses) showing up to 15-fold increase over background at the highest dose of 500 ng (Fig. 2b). Expression of Nsp5 from SARS-CoV-1, MERS or 229E resulted in 7- to 9-fold signal increases at the highest dose, which was consistent with the lower efficiency of ACE2-Gal4 reporter cleavage observed in western blotting of cell lysates (Fig. 2c). ccCov Nsp5s efficiently cleaved and activated the reporter despite differences in expression, as shown by normalization of luciferase signal to the detected Nsp5 protein levels (Fig. 2d). The varied expression levels of Nsp5s might reflect differences in protein stability, as expression did not correlate with reporter activation (Fig. 2e). This suggests that proteases from different hCoVs might not be equally active and/or have different substrate preferences, which might be linked to mutations surrounding the Nsp5 cleavage sites of hCoV (Fig. S2). Overall, the activity of all hCoV Nsp5s could be quantified by the Nsp5 reporter, showing a substantial overlap in substrate recognition and cleavage and highlighting the suitability of the developed assay for studying protease activity of diverse hCoVs.

### Nsp5 reporter is compatible with hCoV inactivation procedures and activated by virally expressed Nsp5

**Fig. 3:**
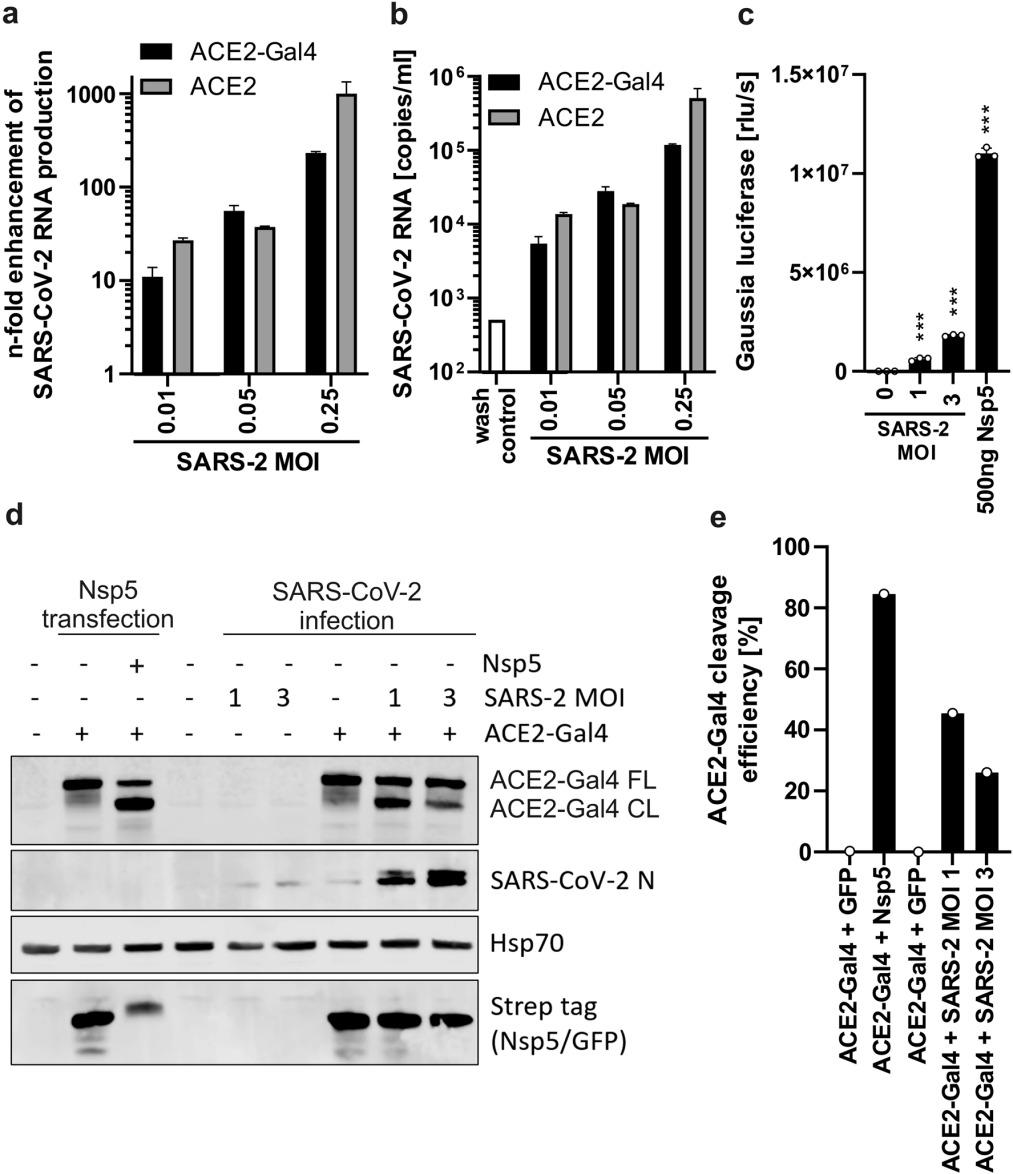
Activation of the Nsp5 reporter assay during hCoV infection. (**a**) Relative levels and (**b**) absolute copy numbers of SARS-CoV-2 RNA present in the supernatant of infected HEK293T reporter cells transfected with plasmid encoding human ACE2 or ACE2-Gal4 fusion protein 48 h after infection. *MOI* multiplicity of infection of input virus. Mean of one independent experiment measured by qRT PCR in technical triplicates, + SD. (**c**) Activation of the Nsp5 Gaussia luciferase reporter assay 18 h after SARS-CoV-2 infection with indicated MOI or by transfection of 500 ng of Nsp5 expression construct. Mean of 3 infections + SD. ***P < 0.001, unpaired Student’s *t*-test. (**d**) Western blot of reporter transfected and infected cell lysates showing cleavage of the ACE2-Gal4 reporter 3 days after SARS-CoV-2 infection (indicated by N staining). Protein concentrations were adjusted prior to loading to account for infection-induced cytotoxicity. Hsp70 served as a protein loading control. (**e**) Quantification of ACE2-Gal4 cleavage efficiency detected by western blot shown in panel (**d**).

During early SARS-CoV-2 infection, ORF1ab, which contains all nonstructural proteins including Nsp5, is transcribed directly from gRNA^32^. To verify if the reporter can be activated by endogenously expressed Nsp5, we measured the reporter activation after hCoV infection. We first confirmed that similarly to wild type ACE2 protein, the ACE2-Gal4 fusion protein promotes SARS-CoV-2 infection of the otherwise poorly-permissive HEK293T cells used in this assay. Infection with low multiplicities of infection (MOI) of SARS-CoV-2 resulted in a dose-dependent increase in viral RNA production at two days post infection (Fig. 3a,b). To test whether the Nsp5 assay is activated by endogenously-expressed viral protease, it was necessary to first evaluate whether the reporter is compatible with any of the temperature, alcohol, and various detergents and fixative-based coronavirus inactivation procedures (Fig. S4A). Gaussia luciferase-containing culture supernatants were unaffected by the tested conditions, with the exception of 4% PFA and 0.5% SDS treatment (Fig. S4B,C). We therefore sought to evaluate the performance of the reporter assay following 30 min sample inactivation at 65 °C.

Reporter cells were transfected with the ACE2-Gal4 reporter and Strep-tagged GFP or SARS-CoV-2 Nsp5 plasmid. After 12 h when GFP expression could be observed, cells were infected with SARS-CoV-2 at MOIs of 1 and 3. Supernatant samples were harvested at 18 h post infection before any cytopathic effects could be observed and inactivated at 65 °C for 30 min and Gaussia luciferase activity was measured. A significant increase in the Gaussia luciferase signal was observed (Fig. 3c). Cleavage of the ACE2-Gal reporter was also clearly visible in western blot analysis of cellular lysates (Fig. 3d) however it was less pronounced than that observed following the overexpression of Nsp5 protein (Fig. 3e). At the early time point selected for Gaussia measurement (18 h) no cytopathic effects were observed by visual inspection of the cells. In contrast, at the time of cell harvest (3 days post-infection) cytopathic effects of the infection were visible under the light microscope, and the protein concentration of cell lysates had to be adjusted to compensate for the cell loss. Notably, ACE2-Gal4 reporter cleavage was also observed after infection with the ACE2-utilizing NL63 CoV but not with hCoVs OC42 and 229E that use other receptors for entry (Fig. S5). These results show that the sensitivity of the Nsp5 reporter assay is unaffected by most virus inactivation procedures and the reporter is cleaved by endogenous Nsp5 expressed by ACE2-utilizing hCoVs.

### Evaluation of viral protease inhibitor activity using the Nsp5 reporter assay

**Fig. 4:**
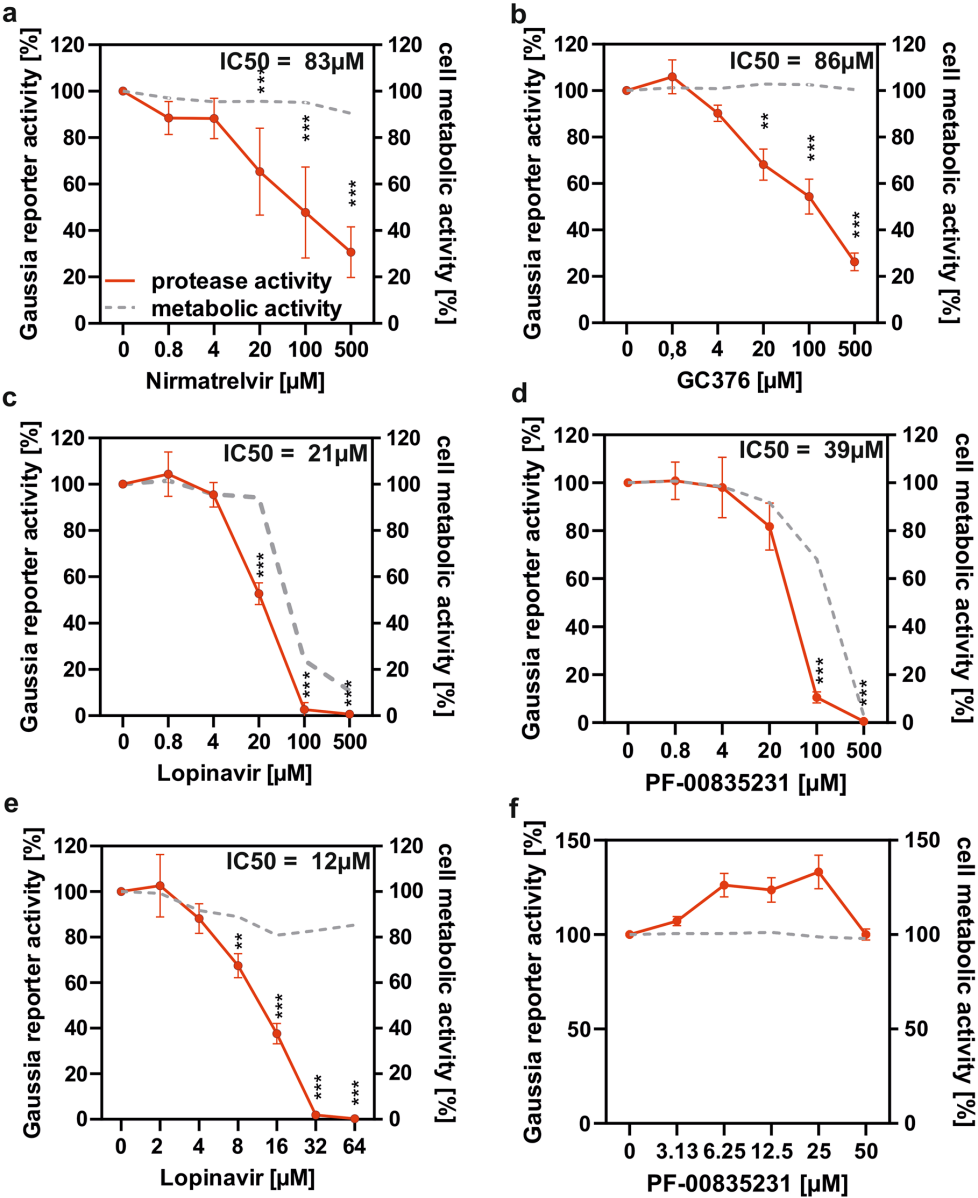
Evaluation of protease inhibitors using the Nsp5 reporter assay. HEK293T reporter cells were co-transfected with equal amounts of plasmid encoding the ACE2-Gal4 fusion protein and SARS-CoV-2 Nsp5 or GFP (negative control). After 6 h cells were treated with indicated molar concentration of inhibitors (**a**) nirmatrelvir, (**b**) GC376, (**c**) Lopinavir and (**d**) PF-00835231 or DMSO control for 20 h. The activity of secreted Gaussia luciferase was measured using luminometer and cellular metabolic activity was evaluated by the MTT assay. The activity of Lopinavir and PF-00835231 the was re-evaluated at lower, nontoxic concentrations (**e** and **f**). Red line indicates relative Nsp5 activity as percentage of no inhibitor control measured by luciferase assay (left y-axis) and grey dotted line indicates metabolic activity as percentage of inhibitor untreated cells as determined by MTT assay (right y-axis). Mean of 3–5 independent experiments + SD. *P < 0.05; **P < 0.01; ***P < 0.001, paired Student’s *t*-test. IC50 values were calculated using Prism GraphPad.

Nirmatrelvir is currently the only widely used coronavirus Nsp5 inhibitor^33^. However, several other protease inhibitors such as GC376, lopinavir and PF-00835231 have been evaluated in vitro and show potential for targeting Nsp5^16–18,34–37^. To verify that the Nsp5 reporter assay can detect the activity of these inhibitors and to compare their relative effectiveness in inhibiting the main protease of SARS-CoV-2, we tested them alongside nirmatrelvir, which served as a positive control. The cells were co-transfected with ACE2-Gal4 reporter and treated with inhibitors 6 h later. Nirmatrelvir and GC376 treatment significantly decreased the Gaussia luciferase signal in Nsp5-transfected HEK293T Gaussia reporter cells at concentrations ≥ 20 µM, with IC_50_ values of 83 µM and 86 µM, respectively (Fig. 4a,b). Lopinavir was also found to significantly inhibit Nsp5 at ≥ 20 µM with an IC_50_ of 21 µM but appeared to be cytotoxic at all of the tested active concentrations (Fig. 4c). For PF-00835231, 100 µM was the lowest tested dose that significantly inhibited Nsp5 reporter activity, whereby the IC50 was estimated by interpolation to be 39 μM; however, this and higher doses strongly reduced cell viability (Fig. 4d). The low specific activity of this compound could be due to the efflux transporter P-glycoprotein, which is highly expressed in HEK293T cells and was previously reported to diminish the effects of PF-00835231^21,38^. To determine if lopinavir and PF-00835231 inhibit Nsp5 activity at concentrations that do not affect cell viability, additional concentrations were tested. Lopinavir was found to be effective and non-cytotoxic within the 8–64 µM concentration range with an IC50 of 12 µM (Fig. 4e), while PF-00835231 did not have a significant effect on Nsp5 at non-cytotoxic concentrations ≤ 50 µM (Fig. 4f). Overall, the Nsp5 reporter assay detected the activity of known viral protease inhibitors.

### Evaluation of protease activity and inhibition of Nsp5 mutants using the reporter assay

**Fig. 5:**
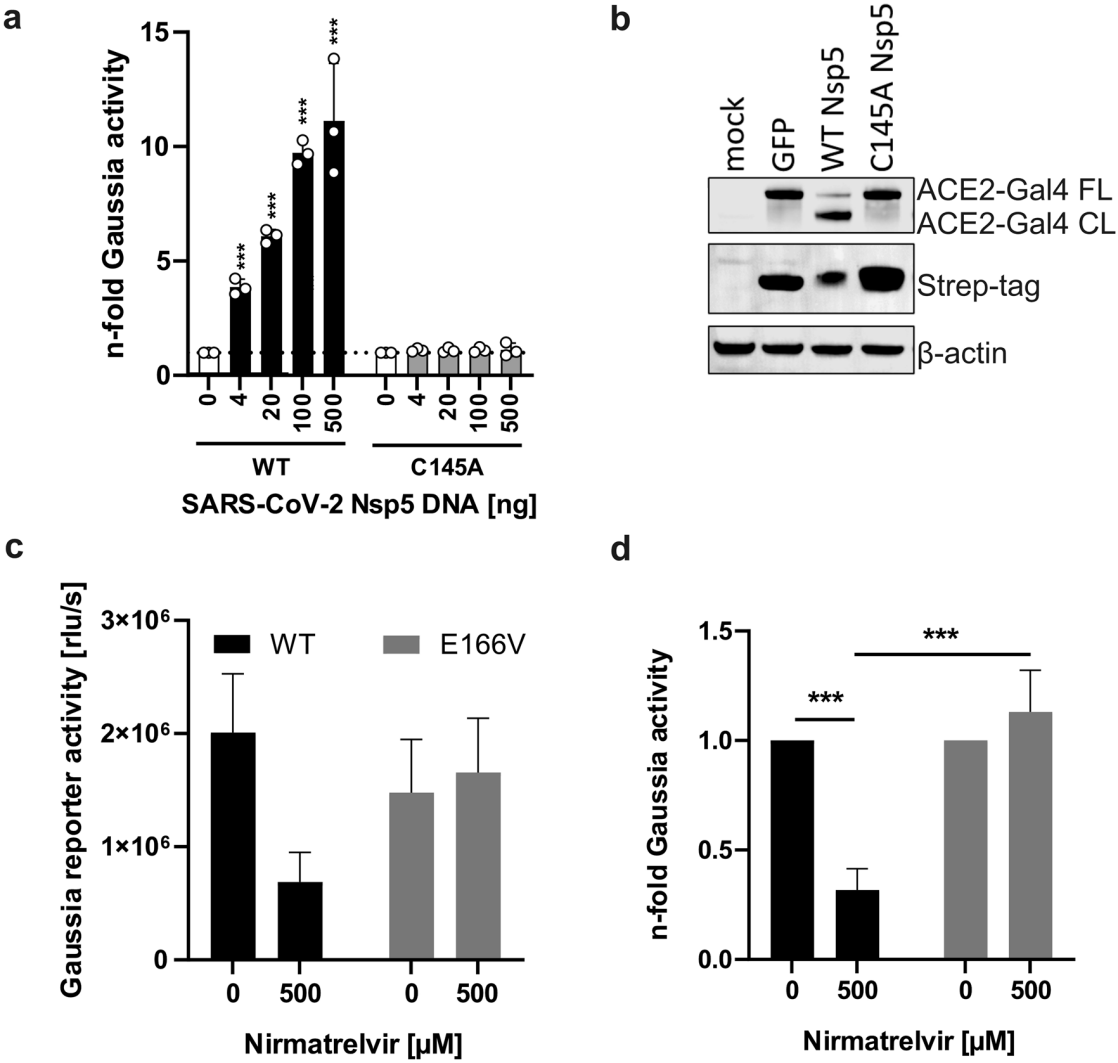
(**a**) Activation of the assay by WT Nsp5 and the C145A catalytically inactive mutant 20 h post transfection. Mean of 3 independent experiments + SD. (**b**) Representative Western blot showing the expression levels of Nsp5 catalytic mutant and its inability to cleave ACE2 reporter. (**c**) Gaussia activity induced by WT Nsp5 protein and E166V Nsp mutant in the presence of nirmatrelvir and (**d**) relative sensitivity of both proteins to the inhibitor. Mean of 3 independent experiments + SD. ***P < 0.001, unpaired Student’s *t*-test.

Since its identification in 2019, SARS-CoV-2 has continued to evolve towards higher transmission, improved immune evasion and increased replication fitness. The properties of emerging SARS-CoV-2 strains need to be monitored to evaluate their virulence and ensure the effectiveness of the employed preventative and therapeutic strategies against COVID-19^33^. To determine if our reporter assay can aid in characterization of Nsp5 variants, we introduced two types of mutations into the Nsp5 expression vector: the C145A substitution known to abolish catalytic activity^39,40^ and E166V associated with increased resistance to nirmatrelvir^8,9,13^. Despite higher expression level, the C145A mutant did not lead to the activation of the Gaussia reporter or cleavage of the ACE2 fusion protein (Fig. 5a and b). In contrast, the E166V mutant was catalytically active (Fig. 5c). Nirmatrelvir treatment inhibited wild type protein activity by 70% but had no impact on the activity of E166V mutant (Fig. 5d). Overall, the developed reporter assay accurately determined the impact of Nsp5 mutations affecting catalytic activity and drug sensitivity.

## Discussion

We developed a reporter assay for fast and efficient evaluation of hCoV protease Nsp5 (3CLpro) activity, which is among the most promising targets for treating coronavirus infections^34^. Our assay allows simultaneous quantification of Nsp5 activity and cell metabolic activity within 24 h, and can facilitate the discovery and evaluation of coronavirus protease inhibitors in the future. Whereas the currently most widely used COVID-19 treatment Paxlovid already targets Nsp5, the development of further inhibitors is warranted due to the risk of drug resistance^8–10^. Our assay is scalable, requires only a luminescence plate reader, and is compatible with the MTT (3-(4,5-dimethylthiazol-2-yl)-2,5-diphenyltetrazolium bromide) cytotoxicity assay, supporting high-throughput screening and rapid evaluation of drug potency and specificity.

hCoV Nsp5 proteases show 20–50% overall amino sequence diversity but share core structural homology and have similar substrate specificity^31^. Therefore, it is possible to develop broadly active hCoV protease inhibitors and a universal assay to study their activities^27^. We demonstrated that our reporter is compatible with Nsp5 homologs from all seven human coronaviruses as well as with most virus inactivation procedures. Furthermore, the assay has a dynamic range of approximately 10-fold between the lowest and the highest tested dose, making it suitable for reliable quantification of overexpressed enzyme activity. By proof-of-concept assessment of nirmatrelvir, GC376, and lopinavir, we demonstrated the ability of our system to detect SARS-CoV-2 protease inhibitors. Furthermore, we showed that it can reliably detect the impact of mutations affecting catalytic function and nirmatrelvir sensitivity of SARS-CoV-2 Nsp5.

There has been substantial interest in Nsp5 reporter assays since the start of the SARS-CoV-2 pandemic, and several versions of such assays exist, many of which rely on fluorescent Flip-GFP technology^27–29,41–43^. Our luciferase-based assay overcomes several disadvantages of GFP-based reporters, such as their considerable confound by pH, the potential autofluorescence of tested compounds, and photobleaching. Compared to GFP, luciferases offer higher stability and more reliable quantification and have been successfully employed in Nsp5 reporter assays^30,41,44–46^. Furthermore, the secretion of the Gaussia luciferase reporter into cell media offers the additional advantages of non-invasive sampling and long-term sample storage.

While screening for potential Nsp5 inhibitors in a virus-free mode is certainly its most prominent application, our assay could be further optimized towards higher sensitivity for the detection of endogenous Nsp5 activity resulting from actual hCoV infections. This is enabled by the fusion of the Nsp5 cleavage sequence to human ACE2, which serves as major entry receptor for SARS-CoV-1, SARS-CoV-2, and NL63. The resulting receptor-reporter fusion protein effectively promoted SARS-CoV-2 entry into the otherwise poorly permissive HEK293T cells^47^. Notably, the produced Gaussia luciferase signal withstood temperature and disinfectant-based virus inactivation procedures without compromising signal-to-noise ratio. Despite these promising results, the sensitivity of the assay would benefit from further improvement to allow the quantification of endogenous Nsp5 activity. Until higher sensitivity is achieved, we do not recommend its use in the context of a genuine hCoV infection for applications that require a highly sensitive and quantitative measure.

The assay detected Nsp5 activity of all human coronaviruses (SARS-CoV-1, SARS-CoV-2, MERS, NL63, OC43, 229E, and HKU1), supporting rapid testing of inhibitors against both existing and potentially emerging pathogens. This finding also implies that it can be used to compare the drug sensitivity and protease function of different coronavirus lineages and to monitor the impact of Nsp5 and pp1ab mutations in emerging SARS-CoV-2 variants on nirmatrelvir sensitivity. While our assay is currently limited to the detection of inhibitors of Nsp5, its design principle can be reiterated to develop reporter assays for other viral proteases, such as hCoV Nsp3/PLpro. Recent studies reported that SARS-CoV-2 Nsp5 cleaves not only the viral polyprotein 1ab but also cellular factors such as NF-κB Essential Modulator (NEMO), septin and Caspase recruitment domain-containing protein 8 (CARD8) which play roles in immune sensing and cell homeostasis^48–50^. Our assay could be utilized for studying cellular Nsp5 substrates and to evaluate inhibitors that not only directly limit viral replication through blocking pp1ab processing but also prevent the Nsp5 associated cellular dysfunction.

Since drug evaluation performed using Nsp5 overexpression results in a more efficient proteolytic cleavage than observed during virus infection, our IC_50_ values are higher than those measured utilizing a genuine virus in other studies. Furthermore, drug efflux transporters highly expressed in immortalized cell lines can affect active drug concentrations^8,21^ and efflux inhibitors such as CP-100356 might be helpful to counteract those effects during screening^36^. Therefore, active concentrations measured using a reporter system might not accurately reflect active concentrations needed in vivo, as demonstrated by the relatively high IC50 of nirmatrelvir (83 µM) measured in our assay compared to 30 nM to 11 µM reported in the literature^9,11,15,28,29,51^. Furthermore, the pharmacokinetics of antivirals in animal models and patients are influenced by many factors, and inhibitors shown to be active in vitro do not always show effectiveness in vivo. This is highlighted by multiple clinical trials reporting no significant benefit of lopinavir in COVID-19 patients, despite the initial reports of its ability to inhibit Nsp5 in biochemical assays^17,19,52–55^. Therefore, it is advisable to use known inhibitors as controls and confirm the activity of newly discovered inhibitors using independent methods and in the context of a genuine virus infection.

Overall, our findings highlight the application of the developed Gaussia luciferase-based reporter assay for studying coronavirus protease activity. Other potential applications include monitoring the impact of emerging SARS-CoV-2 Nsp5 mutations on proteolytic activity and drug sensitivity. Although already highly versatile in its current form, the assay could be modified to study the function of other viral proteases, as well as the impact of mutations of the Nsp5 recognition site. The safety, compatibility, and broad applicability of this assay make it a valuable tool for efforts aimed at combating current and future coronavirus outbreaks.

## Materials and methods

### Cell lines and culture

HEK293T B0166 Gal4 Gaussia luciferase reporter cells^56,57^ were cultured in Dulbecco’s Modified Eagle Medium (DMEM; Gibco, catalog no. 41965039) supplemented with 10% heat-inactivated fetal calf serum (FCS; Gibco, catalog no. 10270106), 2 mM l-glutamine (Gibco, catalog no. 25030081), 100 units/ml penicillin and 100 μg/ml streptomycin (Thermo Fisher, catalog no. 15140122). Huh7 (human hepatocyte-derived carcinoma cell line), LLC-MK2 (rhesus monkey kidney epithelial cell line) and Vero E6 cells (*Cercopithecus aethiops*-derived epithelial kidney line; ATCC) were grown in DMEM with 10% FCS, 100 U/mL penicillin, 100 μg/mL streptomycin and 2 mM l-glutamine.

### ACE2-Gal4 reporter and hCoV Nsp5 construct cloning

hACE2 was amplified from pLV-EF1a-human ACE2-IRES-puro template (primers: TAGAAGCGCGTAGGCCTTTCTAGACCATGTCAAGCTCTTCCTGGCTCCTT and AAATCCACTTTGAAGACGAGCTACTCCTCCTCCAAAGGAGGTCTGAACATCAT; Biomers.net) while Gal4-p65 fusion protein sequence was amplified from MaMTH C-tagged bait vector (primers: CTCGTCTTCAAAGTGGATTTGGAGGAGGAATGAAGCTACTGTCTTCTAT and TAGTACTCCGGGATCCGAACGCGTTACGTAGAATCGAGACCGAG; Biomers.net). The PCR products were ligated into XbaI/MluI HF digested pCG IRES eGFP vector using Gibson assembly (NEB). The correctness of the final construct (pCG hAEC2-VARLQSGF- Gal4-p65 IRES eGFP) was confirmed by Sanger sequencing (Eurofins Genomics).

Codon-optimized hCoV Nsp5 sequences were synthesized by Twist Bioscience. pLVX-EF1alpha SARS-CoV-2 Nsp5 -2xStrep-IRES-Puro and pLVX-EF1alpha SARS-CoV-2 GFP-2xStrep-IRES-Puro were described before^58^. Strep-tagged ORFs were cloned by Gibson assembly (NEB) into the EcoRI/BamHI sites of pLVX-EF1alpha -2xStrep-IRES-Puro vector using the primers: 2229Efwd: (gaggatctatttccggtgaattcaccatggcagggctgaggaaa), 229Erev: (gggagggagaggggcgggatcctcacttttcaaactg), OC43fwd: (gaggatctatttccggtgaattcaccatgtctggcattgtcaaa), OC43rev: (gggagggagaggggcgggatccctacttttcaaactg), NL63fwd: (gtcgtgaggatctatttccggtgaattcgccgccaccatgtcaggcctgaagaagatg), NL63rev: (gttaggggggggggagggagaggggcgggatcctcatttctcgaactggggatg), HKU1fwd: (gaggatctatttccggtgaattcaccatgagtggcatagtaaaa), HKU1rev: (gggagggagaggggcgggatcctcacttttcaaactg), MERSfwd: (gaggatctatttccggtgaattcaccatgtcaggactggtgaag), MERSrev: (gggagggagaggggcgggatcctcatttctcaaattg), SARS-CoV-1fwd: (gaggatctatttccggtgaattcaccatgagtggcttcaggaaa), SARS-CoV-1rev: (gggagggagaggggcgggatcctcatttttcaaactg). The C145A and E166V mutants were generated using Q5 site-directed mutagenesis (NEB) using the following primers: C145A fwd: gttccgttggttttaacatcgactatgac; C145A rev: cggcagagccgttcagaaatgaaccc; E166V fwd: ggtgtccacgccggtacagatctggaag; E166V rev: ggtagggagtaccatatggtgcatgtagc). The correctness of the final constructs was confirmed by Sanger sequencing (Eurofins Genomics).

### Western blotting

To examine the cleavage of hACE2-Gal4 protein by overexpressed Nsp5 proteins or following hCoV infection, cells were lysed in coimmunoprecipitation (CO-IP) buffer (150 mM NaCl, 50 mM HEPES, 5 mM EDTA, 0.10% NP-40, 0.5 mM sodium orthovanadate, 0.5 mM NaF, protease inhibitor cocktail from Roche). Sample protein concentration was measured using Nanodrop and adjusted across the panel, and the samples were reduced in the presence of β-mercaptoethanol by boiling at 95 °C for 10 min. Proteins were separated in 4% to 12% Bis–Tris gradient acrylamide gels (Invitrogen), blotted onto a polyvinylidene difluoride (PVDF) membrane, and incubated with Strep Tag (Cat#ab76949; Abcam), ACE2 (Cat# sc-24; Santa Cruz Biotechnology), GAPDH (Cat# 607902; BioLegend), Hsp70 (Cat# sc-13119, Santa Cruz Biotechnology) SARS-CoV-2 N (Cat#GTX135357, GenTex), GFP (Cat# ab290, Abcam), anti-β-actin (ab8227; Abcam), (NL63 N (Cat#M.30.HCo.I2D4, Ingenasa), OC43 N (Cat# MAB9012, Millipore) and 229E N (Cat# 40640-T62, SinoBiological) antibodies. Subsequently, blots were probed with IRDye 680RD goat anti-rabbit IgG(H + L) (catalog no. 926-68071; LI-COR), IRDye 800CW goat anti-mouse IgG(H + L) (catalog no. 926-32210; LI-COR) or IRDye 800CW goat anti-rat IgG(H + L) (catalog no. 926-32219; LI-COR) Odyssey antibodies and scanned using a LI-COR Odyssey reader. Please note that in cases where the loaded gel area was smaller than the size of the pre-cut membrane, only a part of the blot was scanned, resulting in some of the blot edges not being visible in uncropped blot images.

### SARS-CoV-2 Nsp5 cleavage site analysis and sequence alignment

The Nsp5 cleavage sites of reference SARS-CoV-2 strain 1ab sequence (GenBank: NC_045512.2), as well as Nsp5 sequences of other hCoVs (NL63: NC_005831.2; 229E: NC_002645.1, OC43: NC_006213.1, HKU1: NC_006577.2, SARS: NC_004718.3, MERS: NC_019843.3) were aligned using Multialign tool (http://multalin.toulouse.inra.fr/multalin/)^59^. Sequence logos were generated using WebLogo creator (https://weblogo.berkeley.edu/logo.cgi)^60,61^.

### Transfection of reporter cells

BOI66 cells (0.3 million per well in 12-well format) were incubated overnight at 37 °C. The following day the cells were transfected with 500 ng ACE2-Gal4 reporter and 0–500 ng of Nsp5 or GFP control DNA using PEI Max or LT1. The medium was changed 5–16 h after transfection to either DMEM only (Nsp5 overexpression experiments), DMEM containing hCoVs (hCoV infection experiments), or DMEM containing different concentrations of inhibitors (for the IC50 titration experiments), as indicated in the figure legends.

### Gaussia luciferase assay

Supernatants from the transfected and or infected cells were harvested at 18–48 h post-transfection unless otherwise stated. Gaussia luciferase activity was measured using luminometer plate reader, 2 s after the injection of the coelenterazine substrate.

### MTT assay

To measure the metabolic activity of reporter cells, Methyl-Thiazolyl blue–Tetrazolium bromide salt (MTT-salt) was added to cells at 1:10 ratio. The plates were incubated at 37 °C for 3 h. Formed salt crystals were dissolved in 1 ml of DMSO/EtOH solution. Absorbance was measured at 490–650 nm using a microplate reader.

### hCoV virus stock generation

The BetaCoV/Netherlands/01/NL/2020 strain was obtained from the European Virus Archive and propagated on Vero E6 cells. Cells were inoculated with the SARS-CoV-2 isolate (MOI of 0.03 to 0.1) in serum-free medium. The cells were incubated at 37 °C. HCoV-229E was obtained from ATCC (VR-740TM) and HCoV-OC43 was obtained from ATCC (CR-1558TM). HCoV-NL63 was propagated on LLC-MK2 cells, and 229E and OC43 were propagated on Huh7 cells as described previously^62^. The virus stocks were harvested when the cytopathic effect (CPE) became apparent. The cells were incubated at 37 °C (SARS-CoV-2) or 32 °C (remaining hCoVs). The virus stocks were centrifuged for 5 min at 1000 × *g* to remove cellular debris, aliquoted, and stored at − 80 °C until further use.

### hCoV infection

BOI HEK293T cells were transfected with ACE2-Gal4 (0.5 µg plasmid, 12-well plate) and 12 h later inoculated with SARS-CoV-2 strain NL-2020, NL63, OC43 or 229E at different MOIs as previously described^63^. SARS-CoV-2 infected cells were incubated at 37 °C and other hCoV infected cells were kept at 32 °C. After 6 h input virus was removed and cells were washed in PBS three times. The last PBS wash was kept as a negative control for qRT PCR (“wash control”). Fresh DMEM medium was added and the supernatants and cell were harvested as indicated in the figure legend.

### qRT PCR

RNA from virus-infected cell supernatants was isolated using QIAamp viral RNA minikit (Qiagen, cat#52904) according to the manufacturer’s instructions. Real-time quantitative PCR (RT-qPCR) was performed as previously described^64^ using TaqMan Fast Virus 1-step master mix (Thermo Fisher, catalog no. 4444436). The sequences of the primers and probe for SARS-CoV-2 N gene were as follows: forward primer (HKU-NF), 5′-TAA TCA GAC AAG GAA CTG ATT A-3′; reverse primer (HKU-NR), 5′-CGA AGG TGT GAC TTC CAT G-3′; probe (HKU-NP), 5′-FAM (6-carboxyfluorescein)-GCA AAT TGT GCA ATT TGC GG-TAMRA (6-carboxytetramethylrhodamine)-3′. Primers were purchased from Biomers (Ulm, Germany). Synthetic SARS-CoV-2 RNA (Twist Bioscience, catalog no. 102024) was used as a quantitative standard to obtain viral copy numbers. All reactions were run in triplicate.

### TCID50 assay

To determine the infectious titers of hCoV-229E, hCoV-NL63, and hCoV-OC43, 10,000 Huh-7 cells or Caco-2 (SARS-CoV-2) were seeded 1 day before infection in a 96-well plate. The following day, cells were inoculated with a tenfold serial dilution of the respective virus stock. 4–7 days after infection, cytopathic effects were observed by light microscopy and the tissue culture infectious dose 50 (TCID50) was calculated according to Reed-Münch method^65^.

### Statistical and IC50 analysis

Statistical analysis was performed using GraphPad Prism software. Two-tailed Student’s *t*-test were used to determine statistical significance. Significant differences are indicated as: *p < 0.05; **p < 0.01; ***p < 0.001. The statistical parameters used are specified in the figure legends. IC50 values were calculated using GraphPad Prism software (non-linear fit [Inhibitor vs. response] option).

## Supplementary Information

Supplementary Figures.

## Data Availability

The primary datasets generated and analyzed during the study that are not included in the supplementary material are available from the corresponding author upon request.
