# Supplementary material for: A Gaussia luciferase reporter assay for the evaluation of coronavirus Nsp5/3CLpro activity
Source: Sci Rep. 2024 Sep 5;14:20697. doi: 10.1038/s41598-024-71305-6 (PMC11377810; doi:10.1038/s41598-024-71305-6)
Supplement: Supplementary file 1 — Supplementary Figures. [file 41598_2024_71305_MOESM1_ESM.pdf]

Figure S1. Kinetics of the reporter assay 18-48h post transfection. Related to Fig.1. (A) Non-normalized luciferase values of results shown in Fig.1E. (B) Luciferase signal kinetics. The numbers above bars indicate n-fold difference between 0 (GFP + reporter only) and 500ng Nsp5 construct at each of the tested time points. The 18h and 20h timepoints were selected for subsequent experiments due to best signal-to-noise ratio. Mean of one independent experiment tested in triplicates + SD.

Figure S2. Cell metabolic activity measured using MTT assay. Related to Fig.2. Cells were transfected with 500ng of hCoV Nsp5 proteins and incubated for 24, 48 or 72h. Mean of three independent experiments tested in triplicates + SD.

Figure S3. 3CLpro recognition site of the hCoV 1ab polyprotein. Related to Fig.2. Sequence logos of Nsp5 recognition site are based on 1ab polyproteins of reference hCoV. hCoV panel is the consensus of all hCoV Nsp5 cleavage sites.

Figure S4. Impact of temperature, alcohol and detergent treatment on Gaussia luciferase stability. Related to Fig.3. (A) Experimental setup of results shown in (B – raw data) and (C – normalized data). Supernatants produced as shown in Fig.1E-F were subjected to 30 min treatment and Gaussia activity was measured as described in Fig.1. Mean of 3 independent experiments + SD. *, $P < 0.05$; **, $P < 0.01$; ***, $P < 0.001$, paired Student's t-test.

Figure S5. Cleavage of the ACE2-Gal4 reporter following infection with hCoV NL63, OC43 and 229E. Related to Fig. 3. Cells were harvested 3 days post infection with MOI 3, 1, 0.3 or 0.1 of indicated virus. Cleavage of the ACE2-Gal4 reporter is indicated by the appearance of a second lower ACE2 band, and viral infection is shown by nucleocapsid staining. GFP served as a transfection control and GAPDH served as a protein loading control.

Figure S6. Uncropped western blot scans shown in Figure 1g.

Figure S7. Uncropped western blot scans shown in Figure 2c.

Figure S8. Uncropped western blot scans shown in Figure 3c.

Figure S9. Uncropped western blot scans shown in Figure 5.

Figure S10. Uncropped western blot scans shown in Figure S4.

Figure S1

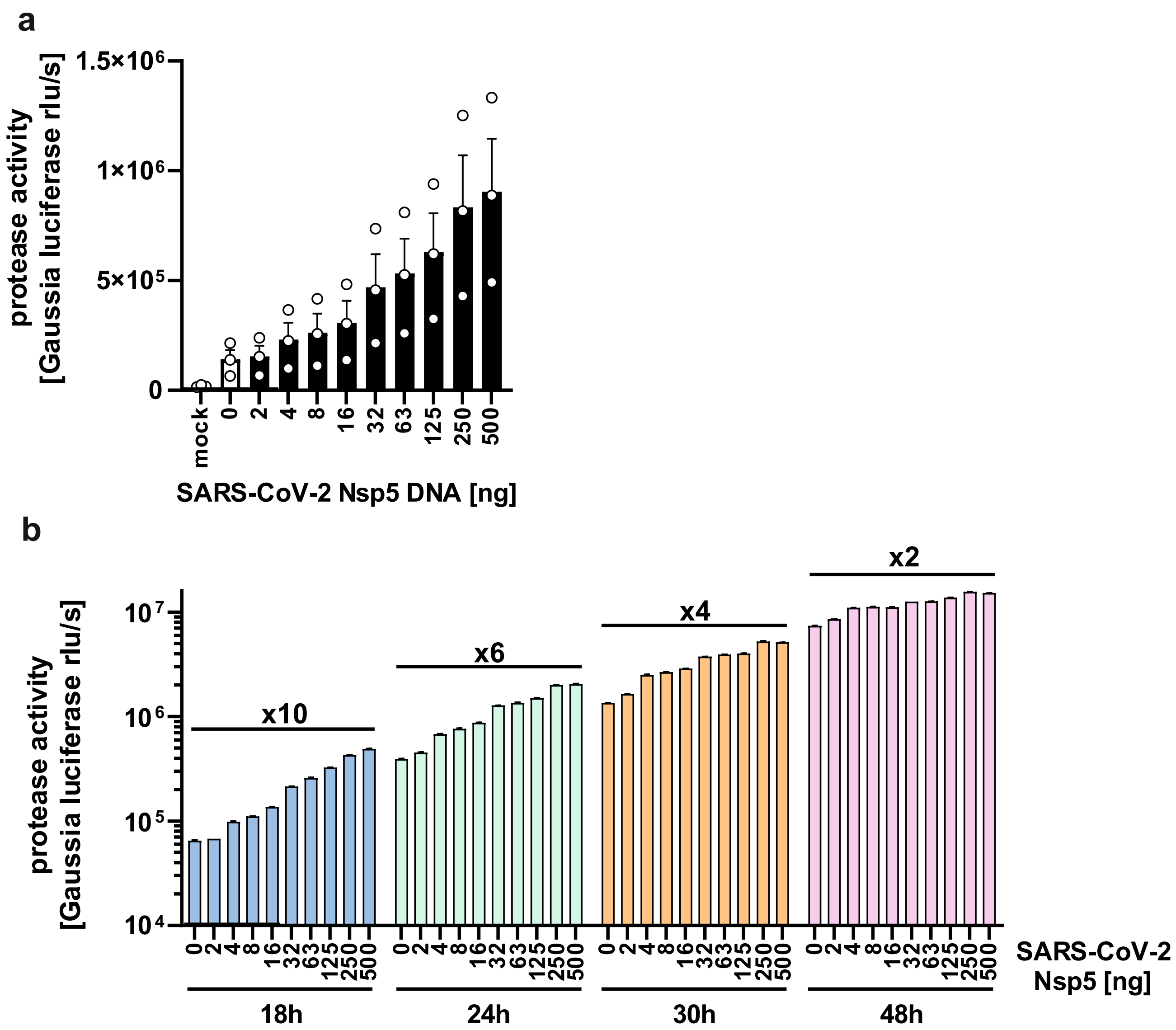

a
protease activity
[Gaussia luciferase rlu/s]
1.5×10⁶
1×10⁶
5×10⁵
0
mock
0
2
4
8
16
32
63
125
250
500
SARS-CoV-2 Nsp5 DNA [ng]
b
protease activity
[Gaussia luciferase rlu/s]
x10
x6
x4
x2
18h
24h
30h
48h
SARS-CoV-2
Nsp5 [ng]

Figure S2

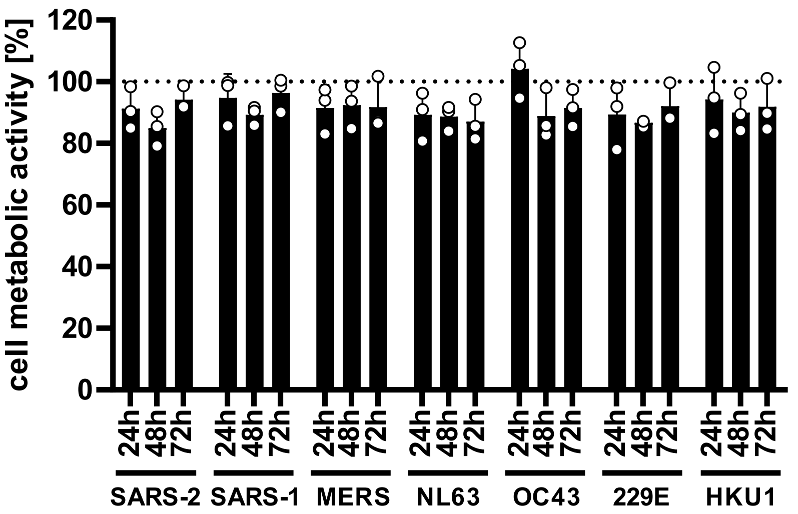

cell metabolic activity [%]
120
100
80
60
40
20
0
24h 48h 72h
SARS-2
SARS-1
MERS
NL63
OC43
229E
HKU1

Figure S3

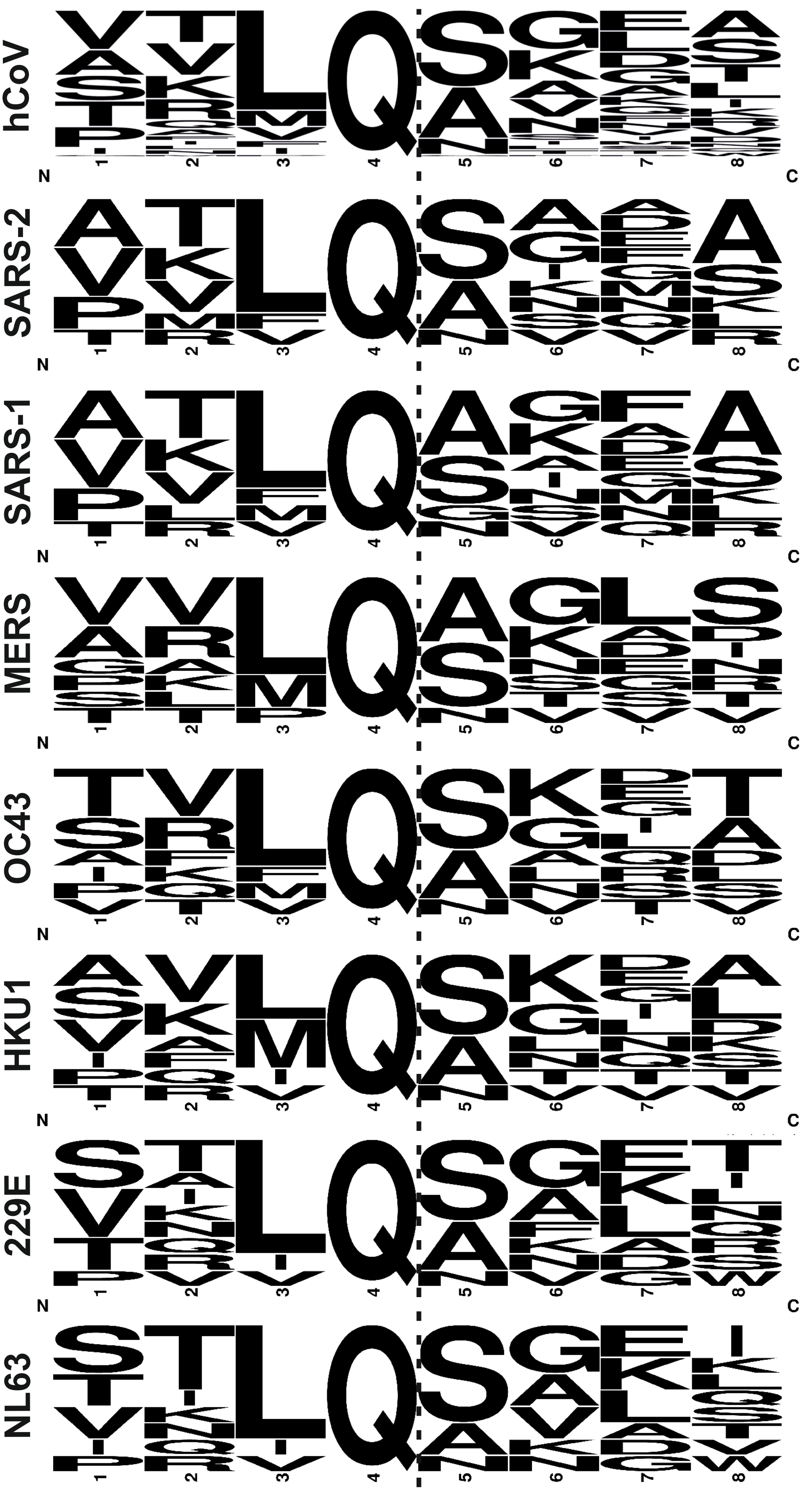

Figure S4

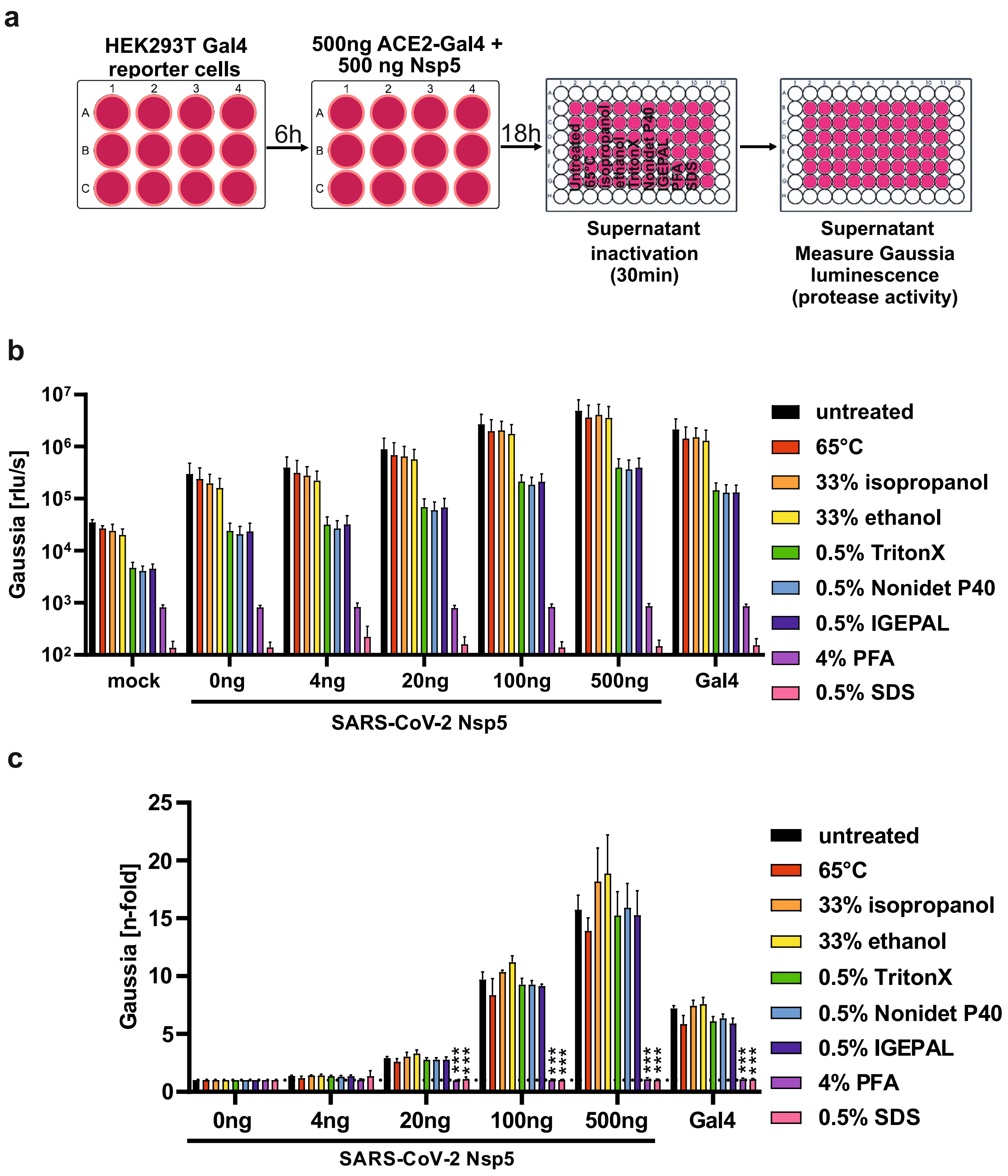

a
HEK293T Gal4 reporter cells
500ng ACE2-Gal4 + 500 ng Nsp5
6h
18h
Untreated
65°C
isopropanol
ethanol
TritonX
Nonidet P40
IGEPAL
PFA
SDS
Supernatant inactivation (30min)
Supernatant Measure Gaussia luminescence (protease activity)
b
Gaussia [rlu/s]
mock
0ng
4ng
20ng
100ng
500ng
Gal4
SARS-CoV-2 Nsp5
untreated
65°C
33% isopropanol
33% ethanol
0.5% TritonX
0.5% Nonidet P40
0.5% IGEPAL
4% PFA
0.5% SDS
c
Gaussia [n-fold]
0ng
4ng
20ng
100ng
500ng
Gal4
SARS-CoV-2 Nsp5
untreated
65°C
33% isopropanol
33% ethanol
0.5% TritonX
0.5% Nonidet P40
0.5% IGEPAL
4% PFA
0.5% SDS

Figure S5

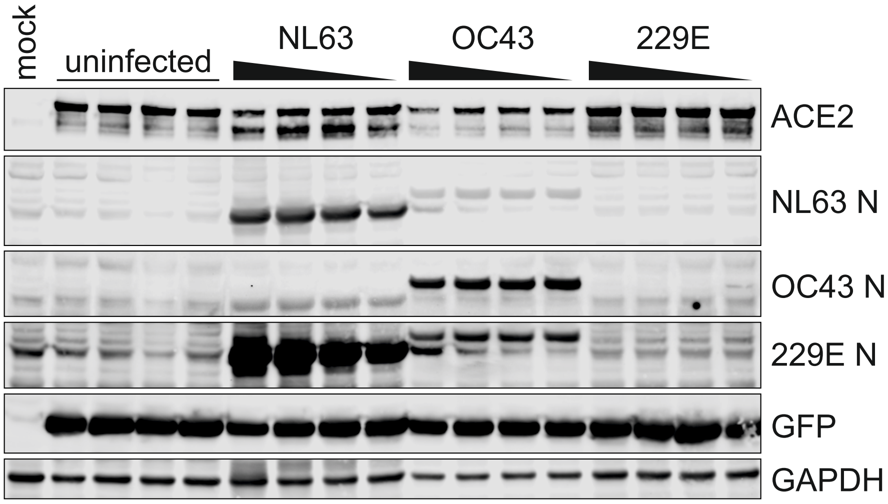
mock
uninfected
NL63
OC43
229E
ACE2
NL63 N
OC43 N
229E N
GFP
GAPDH

Figure S6

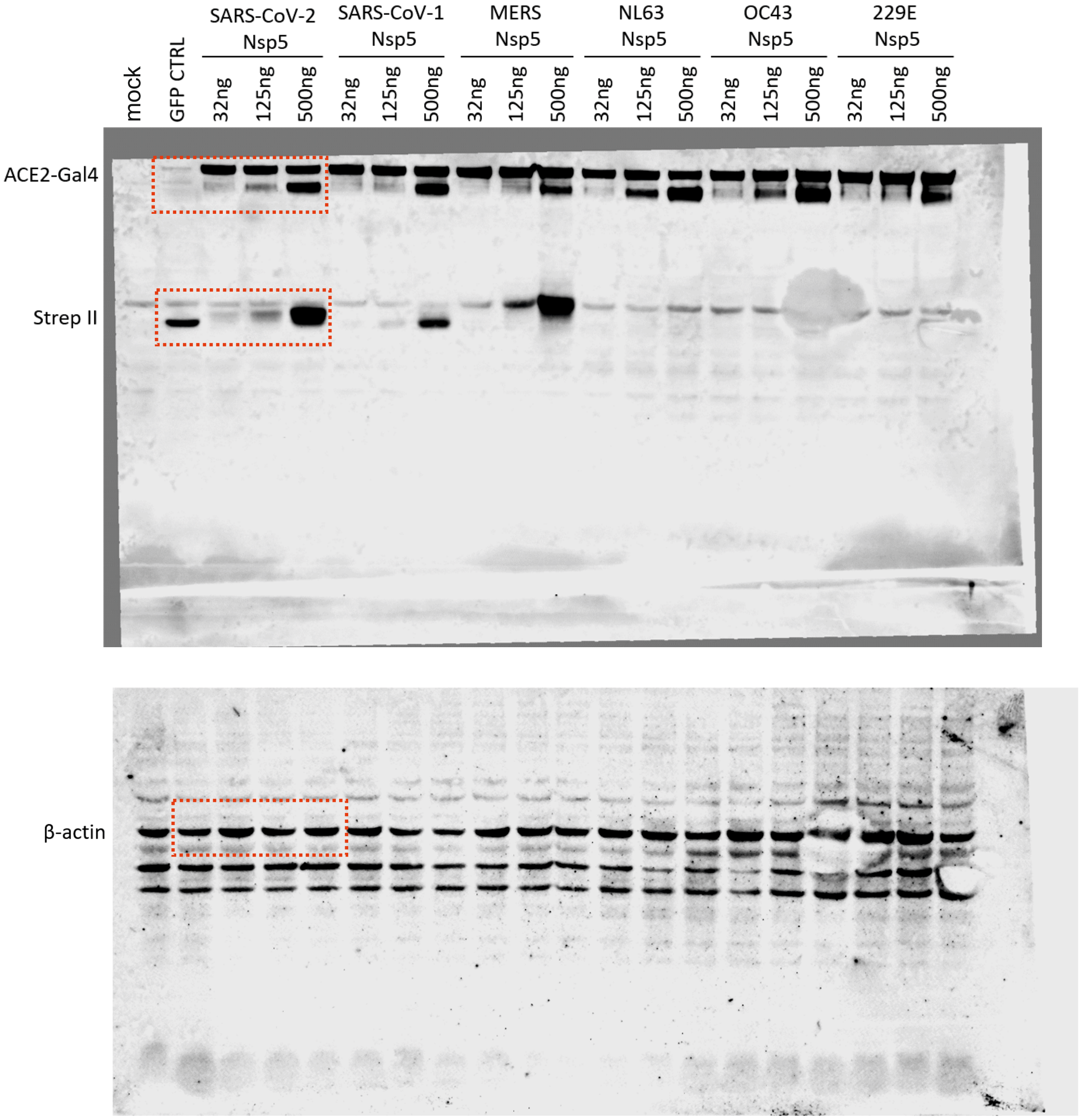

mock
GFP CTRL
SARS-CoV-2 Nsp5
SARS-CoV-1 Nsp5
MERS Nsp5
NL63 Nsp5
OC43 Nsp5
229E Nsp5
32ng
125ng
500ng
ACE2-Gal4
Strep II
β-actin

Figure S7

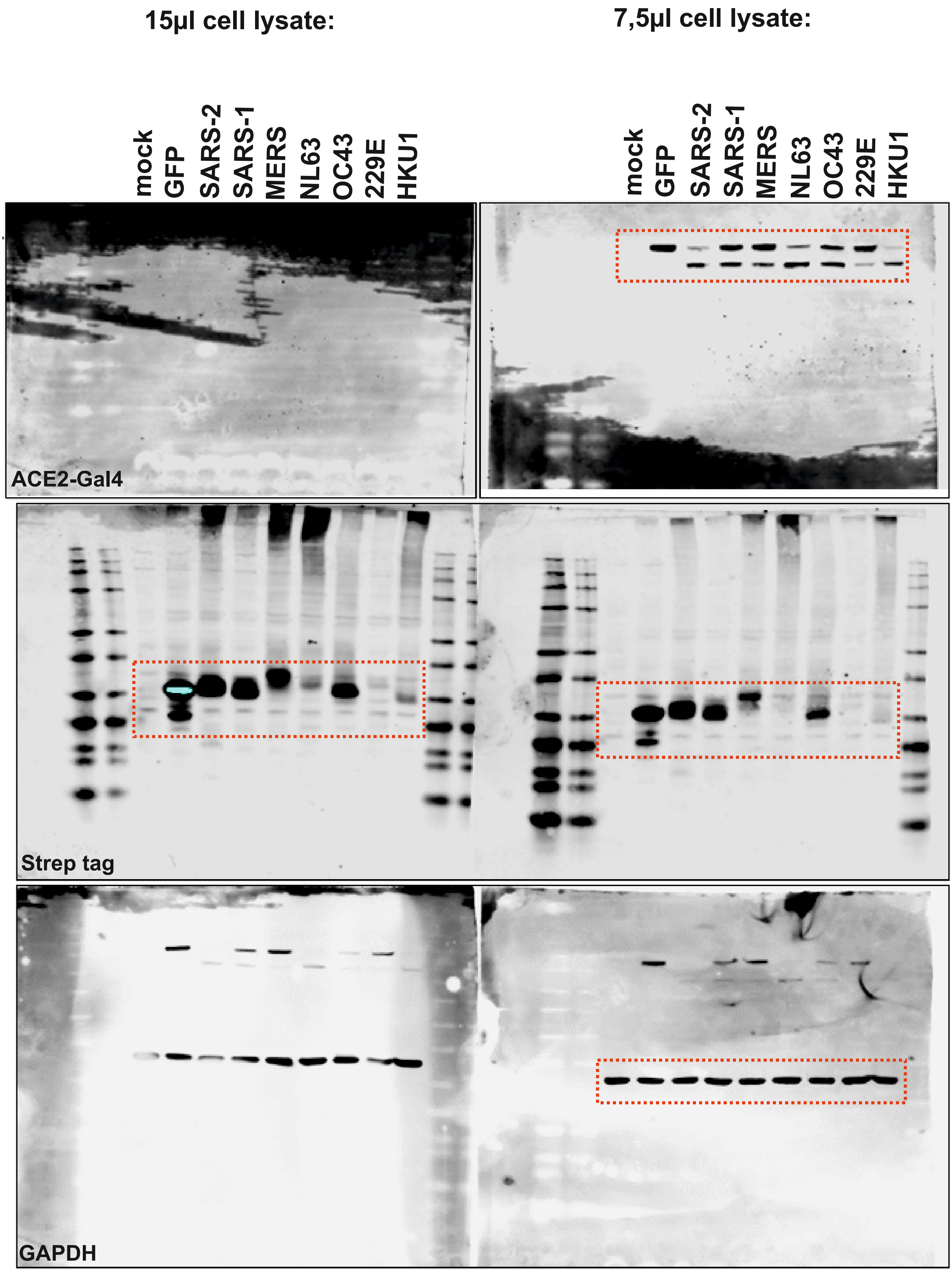
15µl cell lysate:
7,5µl cell lysate:
mock
GFP
SARS-2
SARS-1
MERS
NL63
OC43
229E
HKU1
mock
GFP
SARS-2
SARS-1
MERS
NL63
OC43
229E
HKU1
ACE2-Gal4
Strep tag
GAPDH

Figure S8

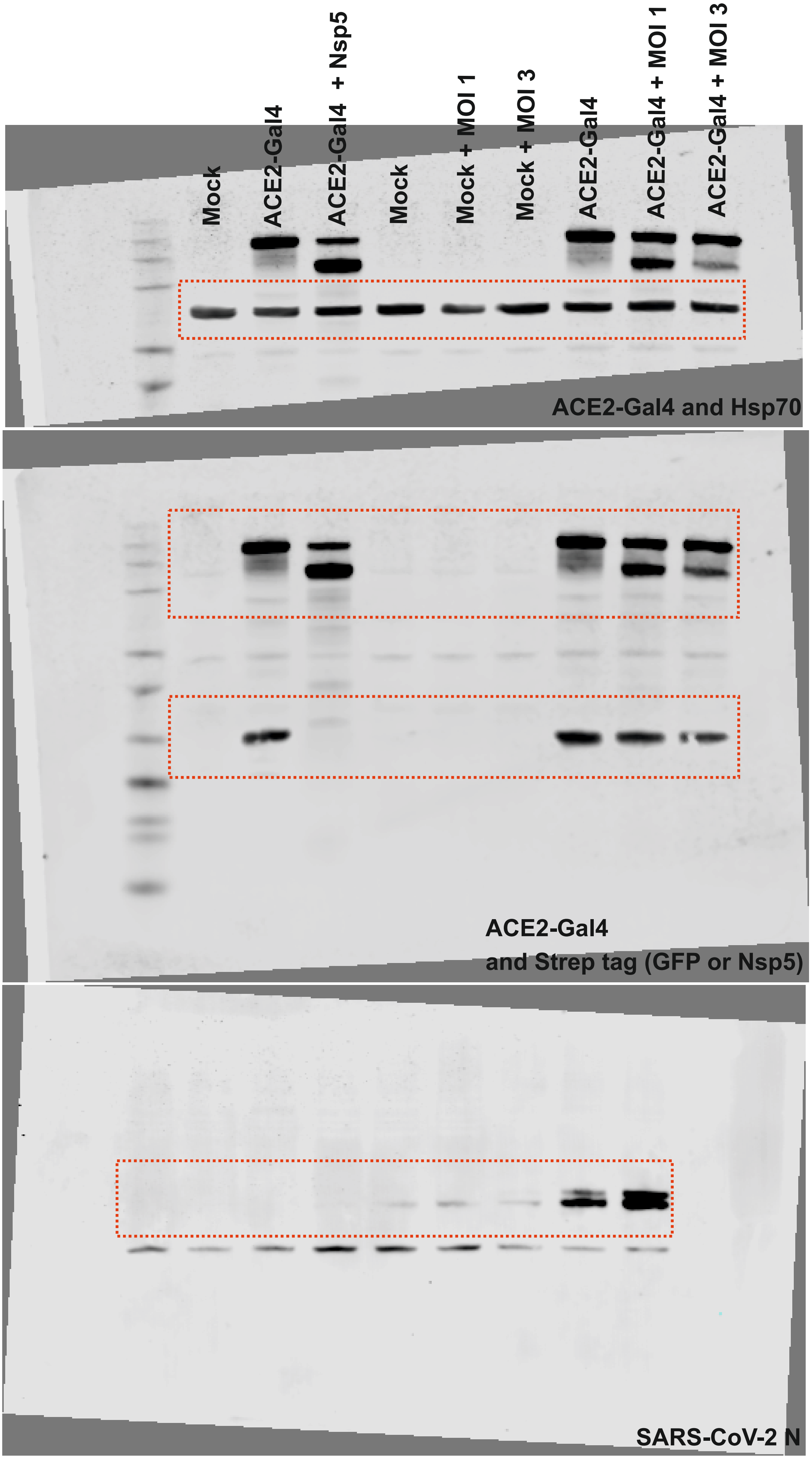

Mock
ACE2-Gal4
ACE2-Gal4 + Nsp5
Mock
Mock + MOI 1
Mock + MOI 3
ACE2-Gal4
ACE2-Gal4 + MOI 1
ACE2-Gal4 + MOI 3
ACE2-Gal4 and Hsp70
ACE2-Gal4
and Strep tag (GFP or Nsp5)
SARS-CoV-2 N

Figure S9

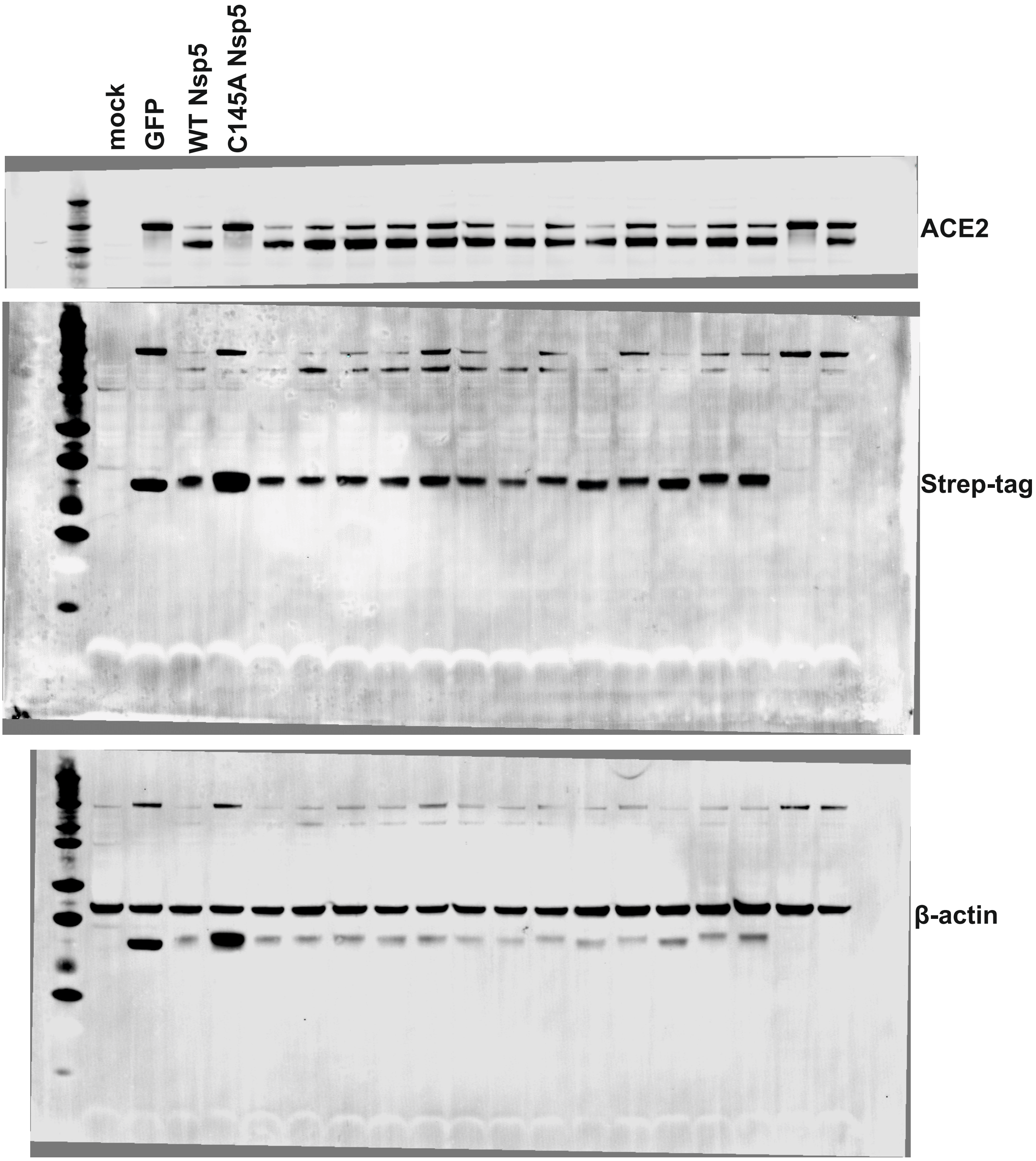

Figure S10

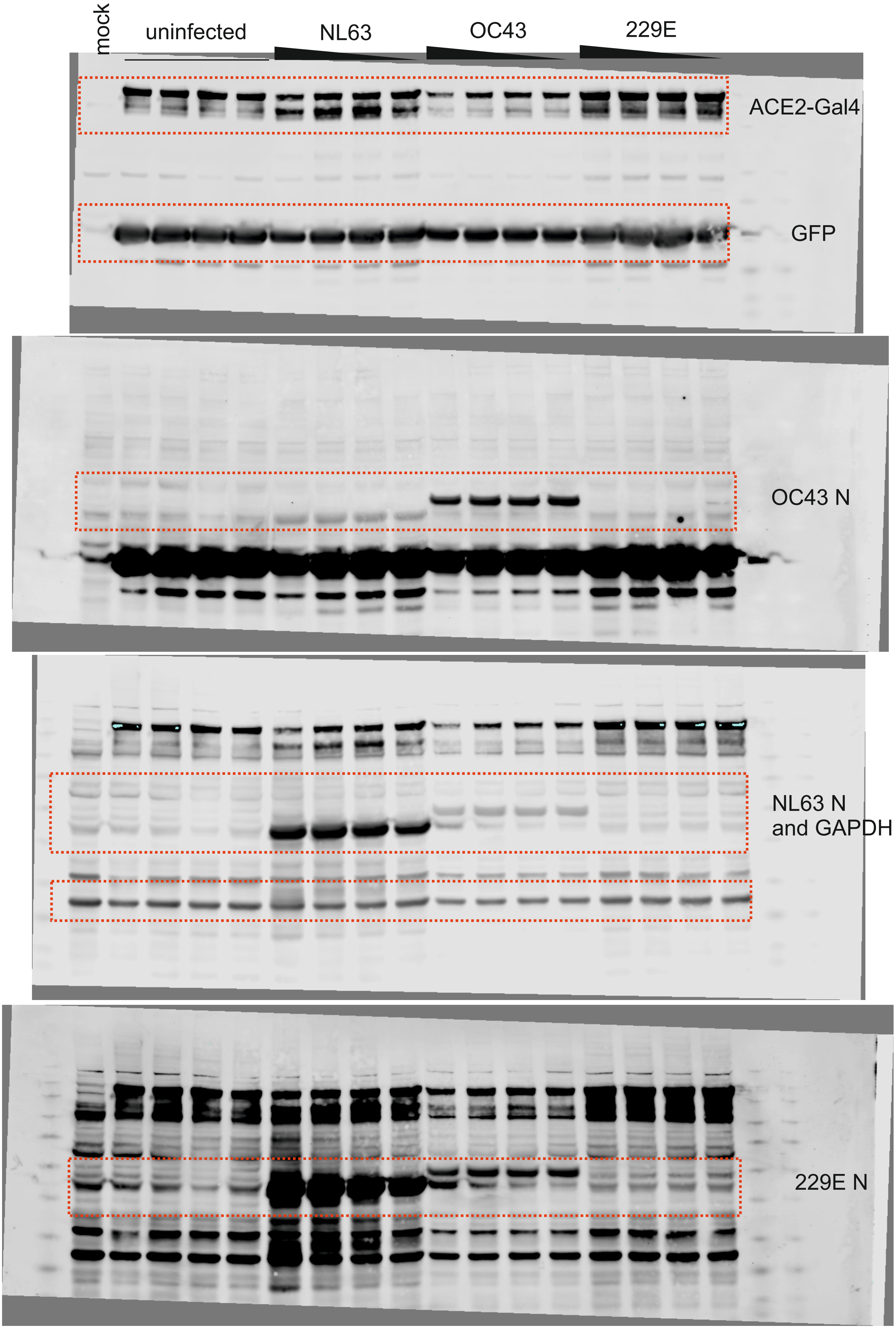
mock
uninfected
NL63
OC43
229E
ACE2-Gal4
GFP
OC43 N
NL63 N
and GAPDH
229E N
